# *In Utero* HIV Exposure and the Early Nutritional Environment Influence Infant Neurodevelopment: Findings from an Evidenced Review and Meta-Analysis

**DOI:** 10.3390/nu12113375

**Published:** 2020-11-02

**Authors:** Marina White, Kristin L. Connor

**Affiliations:** Department of Health Sciences, Carleton University, Ottawa, ON K1S 5B6, Canada; Marina.white@carleton.ca

**Keywords:** HIV, neurodevelopment, nutrition, breastfeeding

## Abstract

The developing brain is especially vulnerable to infection and suboptimal nutrition during the pre- and early postnatal periods. Exposure to maternal human immunodeficiency virus (HIV) infection and antiretroviral therapies (ART) *in utero* and during breastfeeding can adversely influence infant (neuro) developmental trajectories. How early life nutrition may be optimised to improve neurodevelopmental outcomes for infants who are HIV-exposed has not been well characterised. We conducted an up-to-date evidence review and meta-analysis on the influence of HIV exposure *in utero* and during breastfeeding, and early life nutrition, on infant neurodevelopmental outcomes before age three. We report that exposure to maternal HIV infection may adversely influence expressive language development, in particular, and these effects may be detectable within the first three years of life. Further, while male infants may be especially vulnerable to HIV exposure, few studies overall reported sex-comparisons, and whether there are sex-dependent effects of HIV exposure on neurodevelopment remains a critical knowledge gap to fill. Lastly, early life nutrition interventions, including daily maternal multivitamin supplementation during the perinatal period, may improve neurodevelopmental outcomes for infants who are HIV-exposed. Our findings suggest that the early nutritional environment may be leveraged to improve early neurodevelopmental trajectories in infants who have been exposed to HIV *in utero*. A clear understanding of how this environment should be optimised is key for developing targeted nutrition interventions during critical developmental periods in order to mitigate adverse outcomes later in life and should be a priority of future research.

## 1. Introduction

Maternal human immunodeficiency virus (HIV) infection has profound effects on maternal physiology, fetoplacental development, and pregnancy outcomes. Globally, targeted public health programmes and the increasing availability of antiretroviral therapies (ART) provided 85% of 1.3 million pregnant women living with HIV access to the treatments necessary to prevent mother-to-child transmission (PMTCT) in 2019 [1]. As a result, the number of children born annually who are HIV-exposed (*in utero* and during breastfeeding) but are uninfected (HEU) themselves is rising [2]. It is estimated that there are currently 15.2 million children globally who are HEU [2]. The extent to which exposure to maternal HIV infection may have lasting impacts on the development of children who are HEU and the influence of other decisive exposures, including early life nutrition, on these outcomes remains to be thoroughly explored. This gap in understanding limits the development and use of early interventions to improve neurodevelopmental trajectories, tailored to support developmental susceptibilities that children who are exposed to HIV *in utero* or during breastfeeding may experience.

Importantly, persistent immune dysfunction and inflammation experienced by pregnant women living with HIV on ART [3] may heighten offspring risk of neurodevelopmental disorders [4]. Maternal prenatal circulating levels of inflammatory markers associate with the organization of neural networks in the newborn brain, namely in regions critical for attentive abilities, social behaviour, communication, and motor coordination, and are predictive of working memory abilities at two years of age [5]. The developing brain is also vulnerable to the effects of suboptimal maternal nutrition, as nutrition supplied by the mother, prenatally through transplacental transfer and postnatally through breastfeeding and other enteral feeds, supports rapid fetal and neonatal brain development [6]. Breastfeeding is recommended for women living with HIV who are on ART, particularly where undernutrition, diarrhea, and pneumonia are common causes of infant mortality [7], and associates with reduced hospitalization in infants who are HEU during their first year of life [8]. It is critical to understand whether breastfeeding may be beneficial for neurodevelopment in infants who are HEU, as this could provide a target for early nutrition-related interventions.

It is also necessary to consider interactions between exposure to infectious disease (such as HIV) and malnutrition, given that these exposures often coexist in socially inequitable contexts. For example, individuals living with HIV are vulnerable to food insecurity as a result of decreased economic capacity, and food insecurity has been associated with reduced care access and poorer clinical outcomes for people living with HIV [9]. Maternal immunosuppression related to HIV infection may also be exacerbated by malnutrition [10], and the comorbidity of these exposures for infants *in utero* and during the breastfeeding period may be more detrimental than the occurrence of one of these circumstances alone. The multiple intersections of HIV/AIDS and food insecurity have led to calls for integrated nutrition and HIV/AIDS programming [11].

Previous reviews have reported poorer neurodevelopmental outcomes in school-aged children who are HIV-exposed and perinatally infected (HEI) [12,13], and HEU may have persistent, negative effects on neurodevelopment until at least age eight [14]. However, to our knowledge, there has been no review and meta-analysis of evidence related to early life nutritional exposures and neurodevelopmental outcomes in infants who are HEI or HEU. Nutritional factors likely contribute to these outcomes, as vitamin A and macronutrient supplementation have been linked to reduced mortality and improved growth outcomes, respectively, among children who are HEI [15], and nutritional status is a critical determinant of early neurodevelopment [6]. Prenatal folic acid, calcium, and multivitamin supplementation interventions have successfully improved infant development, while vitamin A supplementation and promotion of exclusive breastfeeding have proved effective postnatally [16]. Similar early nutritive interventions may prove effective for infants who are HEU and may be even more vulnerable to the programming effects suboptimal nutrition *in utero* and postnatally [2]. An improved understanding of these relationships is key to optimising early interventions for maximal, positive impact on neurodevelopment and function [6], allowing children to thrive.

Here, we aimed to answer how, and to what extent, do HIV exposure and early life nutritional factors during critical windows of brain development influence infant neurodevelopmental outcomes. Specifically, we first synthesised evidence on how exposure to maternal HIV infection *in utero* and during breastfeeding affects the neurodevelopmental outcomes of infants who are HEI or HEU in the first three years of life. Next, we investigated how early life nutritional exposures (breastfeeding practices, nutrition-related interventions, and food security circumstances) may modify the developmental trajectories of these infants. We also examined sex differences in neurodevelopment and how early life nutrition factors may influence these outcomes for infants who were exposed to HIV, given that male infants are often more susceptible to developmental insults experienced *in utero* in comparison to female infants [17].

## 2. Materials and Methods 

### 2.1. Inclusion Criteria 

Article screening took place as part of a larger scoping review, inclusive of papers relating to growth, immunity, and neurodevelopmental outcomes in infants who are HEI or HEU, and the influence of early life nutritional factors on these outcomes. The Preferred Reporting Items for Systematic Reviews and Meta-Analyses (PRISMA) reporting guidelines were followed (Supplementary Table S1) [18]. Within the neurodevelopment theme, eligible study designs were randomised controlled trials (RCTs), controlled clinical trials, cohort, case series, case-control, or cross-sectional studies. Articles that included at least one group of infants exposed to maternal HIV infection (either HEI, HEU, or HIV-exposed but infant infection status unknown) who had a neurodevelopmental assessment before three years of age were eligible for inclusion. When available, data on outcomes for infants who were born to mothers who did not have HIV and were not infected themselves (HIV-unexposed, uninfected (HUU)) were also considered for between-group comparisons. Clarity of reporting on the method for infant classification as HEI or HEU was evaluated in the methodological quality assessment (Section 2.5). The age criteria allowed us to capture information on early life development within a relatively focused window, inclusive of the recommended period of exclusive (six months) and mixed-breastfeeding (24 months) [19,20]. The first 36 months (3 years) of life are also an especially sensitive period for neurodevelopment, as the brain’s structure and functional capacity rapidly develops during this time [6,21]. Detecting differences in developmental outcomes before three years of age is critical for determining whether children who are HEI or HEU may benefit from the introduction of additional support during critical developmental periods, and in what areas the support is needed, in order to improve developmental trajectories. Lastly, articles that reported on data collected prior to 2000, when international PMTCT efforts were first launched [22], were excluded from review in an effort to increase comparability across studies and relevance to the current-day context of treatment and management of HIV infection in pregnancy, given the drastic shifts in the global response to HIV over the last 20 years.

### 2.2. Information Sources and Search Terms

PubMed, CINAHL, ProQuest, and Web of Science were used to retrieve peer-reviewed publications on pre-defined key terms (Supplementary Figure S1) to extract papers related to growth, neurodevelopment, and immunological status in infants exposed to maternal HIV infection *in utero* or during breastfeeding. The search yielded a total of 20,642 peer-reviewed articles in the English language, including 16,501 duplicates, which were subsequently excluded (EndNote Web), leaving 4141 articles eligible for level one screening. 

### 2.3. Article Screening and Data Collection

#### 2.3.1. Level One: Screening for Growth, Neurodevelopment, and Immune Outcomes

A three-level screening process was constructed with the inclusion and exclusion parameters set to capture relevant articles (Supplementary Figure S2). References for 4141 articles from the EndNote Library were exported into the Distiller SR software for systematic review. At level one, article titles were reviewed for relevance and classified according to theme (growth, neurodevelopment, or immunological status), resulting in the exclusion 2858 articles. These largely included review articles, articles on policies to prevent mother-to-child transmission of HIV, the socio-cultural impacts of living with HIV, or counselling for mothers living with HIV on breastfeeding practices. Articles that discussed policies on infant vaccination schedules and administration or studies that strictly reported on a country’s mortality, morbidity, and survival trends were also excluded. Where a clear assessment of eligibility based on the article title was not possible, the article was carried forward to level two screening.

#### 2.3.2. Level Two: Screening for Neurodevelopment

In the second level of screening, references for 358 articles related to neurodevelopment and 183 that remained unclassified were extracted for abstract review. At this level, 447 articles were excluded where neurodevelopmental outcomes were not reported in the study, or assessments had not occurred prior to three years of age. 

#### 2.3.3. Level Three: Screening and Data Collection for the Neurodevelopmental Theme

The third level screening included full article review and data collection, for which 94 articles within the neurodevelopmental theme were included. Pre-structured forms within the literature review software captured neurodevelopmental outcomes related to cognition, motor, behaviour, language, and neurostructural development. At this level, articles that indicated a primary exposure of interest other than maternal HIV infection or early life nutritional factors, and that did not report comparisons of neurodevelopmental outcomes based on either of these factors, were excluded. These studies included RCTs investigating relationships between timing of ART initiation or different ART therapies and infant health outcomes, and observational studies reporting on the influence of child-caregiver interactions on infant development among infants exposed to HIV, as these were considered outside of the scope of this review. Lastly, given the time lapse between the initial literature search (during November 2016) and the write up of this review, an additional search was performed by hand on 25 March 2020, using the same set of pre-determined key words in each of the four databases to capture any relevant studies published since the original screen. Eleven additional articles were identified in this secondary search, and in total, 24 articles met full eligibility criteria and were included for evidence review and meta-analysis. 

### 2.4. Screening for Early Life Nutritional Factors Within the Neurodevelopmental Theme

All 24 articles that met the primary inclusion criteria for assessment of neurodevelopmental outcomes were subsequently screened for inclusion of data related to maternal nutritional status, breastfeeding practices or reports of food insecurity during pregnancy and the postpartum period, or infant nutritional status in the first three years of life, for which nine articles met at least one of these criterion. 

### 2.5. Methodological Quality Assessment 

Articles were assessed for methodological quality according to the study design using the following scales: Newcastle–Ottawa Quality Assessment Scale [23], the Quality Appraisal Tool for Case Series (18-item checklist) [24], and the Cochrane Collaboration’s Tool for Assessing Risk of Bias [25] for cohort studies (*n* = 15), case series (*n* = 6), and RCTs (*n* = 3), respectively, as has been previously recommended [26]. Criteria for methodological quality assessment were set a priori for each scale and are described in detail in Supplementary Table S2. In brief, for cohort studies, comparability of exposed and non-exposed cohorts was determined based on whether or not analyses controlled for infant sex and age. Notable differences in neurodevelopment and vulnerability to insult have been recorded for male versus female infants [27], and variation in age at neurodevelopmental assessment between groups was considered a potential confounder [28]. Where neurodevelopmental data were longitudinal, adequacy of follow up cohorts was determined when subjects lost to follow up were minimal (set at <20%), or analyses were run to establish similarity between infants retained at follow up vs. not, as previously recommended [28]. For case series, characteristics of the cohort that were important to report were pre-defined as: number of participants (infants), age range of infants with neurodevelopmental assessments at each time point, and infant sex. Intervention and co-intervention definitions were modified to be exposure of interest (maternal HIV infection and ART) and co-exposure of interest (infant HIV and treatment status). For RCTs, “other bias” was defined as an assessment of participant compliance to intervention. While methodological quality was not a primary outcome of interest in this review, it was deemed necessary to help inform our interpretations and weighing of results across studies. 

### 2.6. Data Analysis

A random effects meta-analysis was performed on data from studies that used the Bayley Scales of Infant Development 3rd ed. (BSID-III) [29] and reported scaled or composite scores for the cognitive sub-scale [30]. A random effects model was chosen because it considers between-study variance [31]. However, it is recommended to have a minimum of five studies when using a random effects model [31], and only three studies reported BSID-III scaled or composite scores for the gross and fine motor, and expressive and receptive language sub-scales. Thus, as we were underpowered to synthesize the results for these four subscales through meta-analyses, we report their combined effect estimates for information’s sake only and discuss these findings qualitatively. Raw scores were not considered in meta-analyses, as they are not age-adjusted. Between-group comparisons for infants who were HEU and HUU were considered. Hedge’s *g* was chosen as an estimate for effect size measurements, as it has been shown to be accurate in the case of small sample sizes [32]. Heterogeneity (*I*^2^) was not calculated, as it has shown to be highly biased in small sample sizes [33]. Statistical significance was confirmed at α = 0.05, and results are presented as Hedge’s *g* (95% confidence interval).

## 3. Results

### 3.1. Study Location, Demographics, and Design

The articles under review included participant data from 17 countries (Supplementary Figure S3). Of the cohorts included in studies under review, 57% (*n* = 17) were from Africa, followed by 17% (*n* = 5) from North America, 13% (*n* = 4) from South America, and 7% (*n* = 2) from both Asia and Europe. One article reported on data from cohorts in Brazil, Argentina, Peru, Mexico, Bahamas, and Jamaica [34]. South African cohorts had the highest representation in studies under review (29%, *n* = 7).

**Figure 1:**
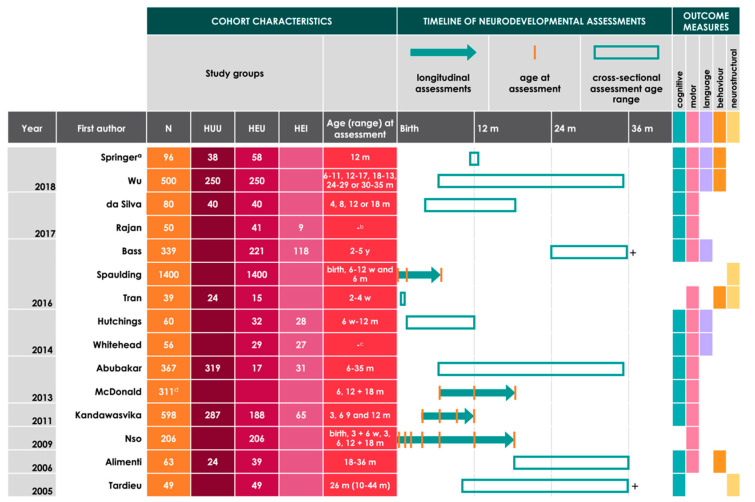
Adapted Graphical Overview for Evidence Reviews (GOfER) diagram [34] of studies reporting on neurodevelopmental outcomes in infants in relation to HIV-exposure status. A plus sign (+) beside the timeline bar indicates that assessments were performed for infants beyond 36 months of age. HUU = HIV-unexposed, uninfected; HEU = HIV-exposed, uninfected; HEI = HIV-exposed, infected; d = days; w = weeks; m = months; y = years. ^a^ Two studies report on data from the Mother and Infant Health Study (MIHS) cohort [48,52]. ^b^ Development was assessed for all children at enrolment (range 6–18 m) and after 3 months. A third assessment was done for 25 children after 6 months of enrolment. ^c^ Infants under one year of age were eligible for recruitment. Neurodevelopment was assessed at baseline (prior to initiating ART for infants who were HEI) and again three and six months later. Breakdown of infant ages at baseline, second, and third assessments was not provided. ^d^ A breakdown according to infant HIV status for the 311 infants who had neurodevelopmental assessments was not available. All were HIV-exposed. One hundred and thirty-nine HEI and 519 HEU assessments were used in analysis for both cognitive and motor outcomes (repeat measures for infants were included). Data from this infant cohort are reported in another study under review [50].

**Figure 2:**
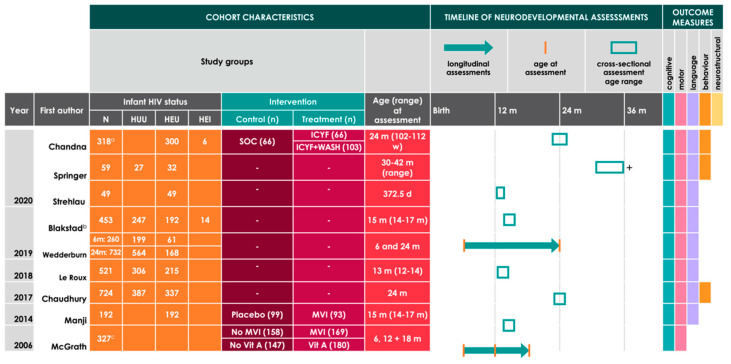
Adapted Graphical Overview for Evidence Reviews (GOfER) diagram [34] of studies reporting on early life nutritional factors and neurodevelopmental outcomes in infants affected by exposure to maternal HIV infection. Detailed descriptions of the study treatments or interventions are presented elsewhere (Supplementary Table S3). A plus sign (+) beside the timeline bar indicates that assessments were performed for infants beyond 36 months of age. HUU = HIV-unexposed, uninfected; HEU = HIV-exposed, uninfected; HEI = HIV-exposed, infected; SOC = standard-of-care; ICYF = infant and young child feeding; WASH = water, sanitation, and hygiene; Vit A = Vitamin A intervention; MVI = Multivitamin intervention; d = days; w = weeks; m = months. ^a^ There were 12 infants included in the analyses whose HIV status was unknown. ^b^ Reports on two infant cohorts who were enrolled in two separate micronutrient trials in Tanzania. One of these cohorts is reported on in another study under review [57]. ^c^ All infants were HIV-exposed. Authors did not report cohort breakdown according to infant HIV status but did report that infant HIV status did not modify relationships between multivitamin supplementation and neurodevelopmental outcomes.

For the 24 studies included for review, cohort characteristics, including study groups, age at neurodevelopmental assessment, timeline of neurodevelopmental assessments (cross-sectional or longitudinal), and outcome themes are reported in Figure 1 and Figure 2 in adapted Graphical Overview for Evidence Reviews (GOfER) diagrams [35]. Studies comparing neurodevelopmental outcomes based on infant HIV exposure status (primary inclusion criteria, *n* = 15) are summarised in Figure 1, and studies that reported on both early life nutrition-related variables and infant neurodevelopment (primary and secondary criteria, *n* = 9) are summarised in Figure 2. Within the 15 studies that met the primary inclusion criteria only, there were six that reported on longitudinal neurodevelopmental outcomes (for cognitive (*n* = 3), motor (*n* = 4), language (*n* = 1), and neurostructural (*n* = 1) themes) for infants before age three [34,36,37,38,39,40], and nine that reported cross-sectional data (for cognitive (*n* = 8), motor (*n* = 8), language (*n* = 4), behavioural (*n* = 4), and neurostructural themes (*n* = 2)) [41,42,43,44,45,46,47,48,49]. For the nine studies that included analyses on early life nutrition-related variables and infant neurodevelopment, cohort characteristics and comparison groups based on nutritional intervention (if relevant) are reported in Figure 2. Within these nine studies, two reported data on longitudinal neurodevelopmental assessments (for cognitive (*n* = 2), motor (*n* = 2), and language (*n* = 1) outcomes) [50,51], and seven reported cross-sectional assessment data (for cognitive (*n* = 7), motor (*n* = 7), language (*n* = 7), and behavioural (*n* = 3) outcomes) [52,53,54,55,56,57,58]. 

### 3.2. Study Measurement Tools

The measurement tools employed to assess neurodevelopmental outcomes are reported in Table 1 and Table 2 for each study. The most common assessment scales were the Bayley Scales of Infant Development—1st to 3rd editions [29,59,60]. To assess neurostructural outcomes, two articles used structural imaging techniques (magnetic resonance imaging and diffusion tensor imaging [44,48] and one article used the World Health Organization standardised growth measures [61] to ascertain microcephaly in infants [34]).

### 3.3. Methodological Quality Assessments

**Figure 3:**
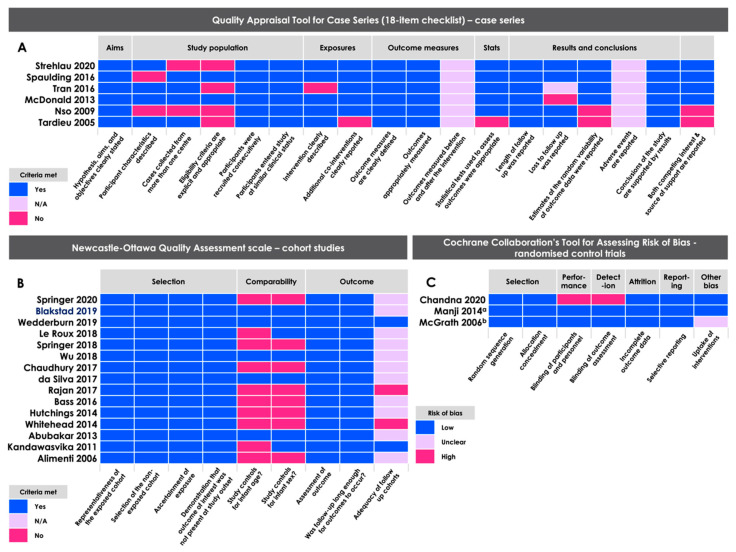
Quality assessment of articles according to study design using the (**A**) Quality Appraisal Tool for Case Series (18-item checklist, case series [23], the (**B**) Newcastle–Ottawa Quality Assessment Scale (cohort studies [22]), and the (**C**) Cochrane Collaboration’s Tool for Assessing Risk of Bias (randomized controlled trials [24]). ^a^ This sub-study was a secondary endpoint of a larger randomised controlled trial. This sample includes children who attended only one of the three research clinics and 12% of the infants who attended follow up at 15 months overall (71.9% of the total number of infants who were randomized). Demographic characteristics across the placebo and multivitamin sub-study groups remained similar, so risk of selection bias (internal) was assessed as low. ^b^ While authors report treatment compliance for the randomized arms, it is not clear that authors considered treatment compliance for the population of mothers whose infants had at least one neurodevelopmental assessment and were included in this analysis (327 of 1078 assigned to treatment arms).

Among the cohort studies under review, assessment criteria were largely met; however, few studies controlled for infant sex and age at assessment (Figure 3A). As 11 of the 15 cohort studies reported on cross-sectional neurodevelopmental measures, evaluating adequacy of follow up cohorts was often not applicable. The case series under review varied in quality, largely in terms of adequacy of participant characteristics, multiple-centre case collection, and clear and appropriate eligibility criteria (Figure 3B). High risk of performance and detection bias was detected in one RCT [52], as it was not possible to blind participants or assessors to the study intervention, given the nature of the treatment (Figure 3C).

### 3.4. Infant HIV Exposure Status Associates with Neurodevelopmental Outcomes in the First 36 Months of Life 

A summary of study characteristics and key findings on neurodevelopmental outcomes from birth to 36 months of age in infants born to mothers living with HIV is presented in Table 1.

#### 3.4.1. Cognitive Outcomes

**Figure 4:**
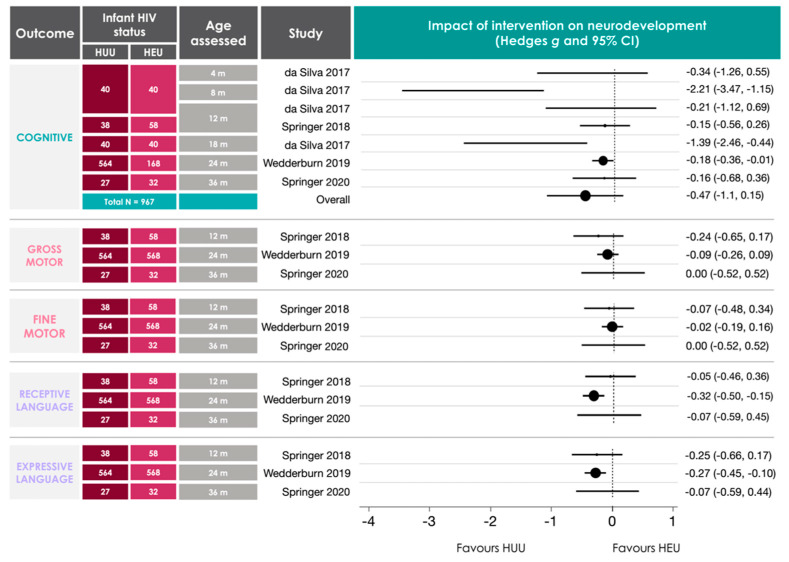
Random-effects meta-analysis for studies reporting on BSID-III sub-scales for infants who are HEU compared to HUU. Studies that reported means and standard deviations for scaled BSID-III scores were included. Where longitudinal data were available, analyses used the oldest data reported to capture any persistent impacts of HEU on neurodevelopment [49]. Data from da Silva et al. (2017) [41] were a cross-section of four separate groups of infants (aged 4, 8, 12, and 18 m). For one study, cognitive data analysed were composite scores, as scaled scores for this domain were not available [48]. Methodological quality assessments for these studies are reported in Figure 3. A summary effect estimate is only included for the cognitive sub-scale, as this is the only assessment that included more than five studies [30]. Data are presented in forest plots as Hedge’s *g* (95% CI) in ascending order according to infant age at assessment. HUU = HIV-unexposed, uninfected; HEU = HIV-exposed, uninfected; m = months; BSID-III = Bayley Scales of Infant Development 3rd edition [28]; CI = confidence interval.

Infants who were HEI frequently scored lower than their HEU counterparts in measures of cognitive development before three years of age [36,40,45] and were twice as likely to exhibit high risk for neurodevelopmental impairment when compared to HEU and HUU infant groups [38]. Reports on cognitive development for infants who were HEU (in comparison to HUU) were often inconsistent, with studies reporting associations between HEU and lower scores on measures of cognitive development [41,42], as well as no difference in scores on measures of cognitive outcomes [49,50,53]. One study reported lower cognitive developmental scores for infants who were HEU compared to HUU; however, these differences did not persist after controlling for maternal substance use [47]. Cognitive developmental delay was often more prevalent among infants who were HEU at 12–13 months [49,56] compared to HUU but may not persist to three years of age [53]. One study reported that in comparison to reference norms, a higher proportion of infants who were HEU had average, higher average, and superior performance on measures of cognitive outcomes [54]. Overall, HEU had a medium, negative effect on BSID-III cognitive subscale scores from four studies (seven cohorts; birth to 36 months) in comparison to HUU; however, the effect was not significant (−0.47 (−1.10, 0.15); Figure 4). A funnel plot illustrating the scatter of effect estimates and standard error for the impact of HEU on infant cognitive outcomes is presented in Supplementary Figure S4. While the symmetry of the funnel suggests a possible negative skew, which could be a result of reporting bias, no test was performed to assess funnel plot asymmetry, as this is not recommended for meta-analyses with less than 10 studies [62].

#### 3.4.2. Motor Outcomes

HEI often associated with lower scores on measures of motor development in comparison to infants who were HEU (up to 35 months of age [36,40,45,46]) and increased likelihood of motor developmental impairment compared to HEU and HUU infant groups [38]. While some studies suggested that infants who are HEU may also experience poorer motor outcomes in comparison to HUU [41,42], others reported no differences in average motor scores between the two groups [47,49,50,53,56] or evidence of motor delay for infants who are HEU [54,56]. One study reported psychomotor developmental delay in 3.64% of their sample (206 HEU) between birth and 18 months in comparison to a cited population rate of 1.1–2.5% [39]. Overall, HEU did not have a significant effect on BSID-III fine (−0.02 (−0.08, 0.04)) or gross (−0.10 ( −0.29, 0.09)) motor subscale scores in three studies (Figure 4) from birth to 36 months, in comparison to HUU. 

#### 3.4.3. Language Outcomes

Of the three studies that reported on language outcomes in HEI infant populations, two reported lower scores [36,45] in comparison to HEU, and one reported no differences [43]. Similar scores between HEU and HUU infant groups were reported for language assessments [53,56] with no evidence of language delay for infants who are HEU [54]. One study reported lower scores and higher odds of delayed development in expressive and receptive language domains at 24 months (but not 6 months) among infants who were HEU compared to HUU in unadjusted and adjusted analyses [50]. Chaudhury et al. also reported lower expressive language scores in infants who were HEU compared to HUU at 24 months; however, the differences were attenuated in adjusted analyses [57]. Overall, HEU had a significant and negative effect on expressive (−0.25 [−0.43, −0.08]), but not receptive (−0.25 [−0.61, 0.10]), language outcomes in comparison to HUU infants, in the first 36 months of life (Figure 4). Importantly, this combined effect estimate largely favours the results of one study, which had a much larger sample size (HUU: *n* = 564, HEU: *n* = 568) [50] than the other two studies (HUU: *n* = 38, HEU: *n* = 58 [49]; HUU: *n* = 27, HEU: *n* = 32 [53]) that were included in this assessment.

#### 3.4.4. Behavioural Outcomes

None of the studies under review assessed behavioural outcomes for infants who were HEI. Overall behavioural scores at 12 months [49] and measures of communication, daily living, and socialization from 18–36 months [47] did not differ between infants who were HEU compared to HUU; however, a higher proportion of HEU infants (12.1 vs. 2.6% HUU) were classified as socially withdrawn [49]. In comparison to HUU infants, one study reported that infants who were HEU had higher scores in measures of personal–social development at 24 months [57], while another reported lower adaptive behaviour scores for infants who were HEU between birth and 35 months [42]. 

#### 3.4.5. Neurostructural Outcomes

Three studies reported on neurostructural development in infants who were HEU [34,44,48], one of which had a HUU comparison group [44]. Microcephaly, defined as a head circumference less than two standard deviations below the average [61], and a risk factor for poorer neurodevelopmental outcomes until at least five years of age [63], was recorded in 7.5% of infants who were HEU (105 of 1400) in one study that reported on data from six countries in Latin America and the Caribbean [34]. At least one neurologic condition (unspecified) was recorded in 9.6% of infants from these cohorts [34]. Of the two studies that characterised neural white matter in infants who were HEU, one recorded high prevalence of diffuse hyperintensity in the tegmentum pons and the supratentorial white matter [48], and one recorded higher fractional anisotropy in the middle cerebellar peduncle region in infants who were HEU compared to HUU [44]. Associations between white matter structural signatures and performance on neurological assessments were also reported [44]. 

### 3.5. Relationships between Early Life Nutritional Factors and Neurodevelopmental Outcomes in Infants Perinatally Exposed to Maternal HIV Infection and ART

A summary of study characteristics and key findings on the influence of nutrition-related factors and interventions on neurodevelopmental outcomes in infants born to mothers living with HIV is presented in Table 2. Infants who were HEU were breastfed at lower rates [57] and for shorter durations [50,56]. Some studies report no associations between breastfeeding practices and neurodevelopmental outcomes up to 36 months [50,53,54] or risk of developmental delay at 13 months [56] in infants who were HEU. One study reported that HEU infants who were ever breastfed had higher cognitive and expressive language scores at 24 months when compared to infants who were never breastfed [57]. Notably, this study also reported associations between household food insecurity and lower gross motor scores, irrespective of maternal HIV status; however, higher rates of food insecurity were reported among infants who were HEU. 

**Figure 5:**
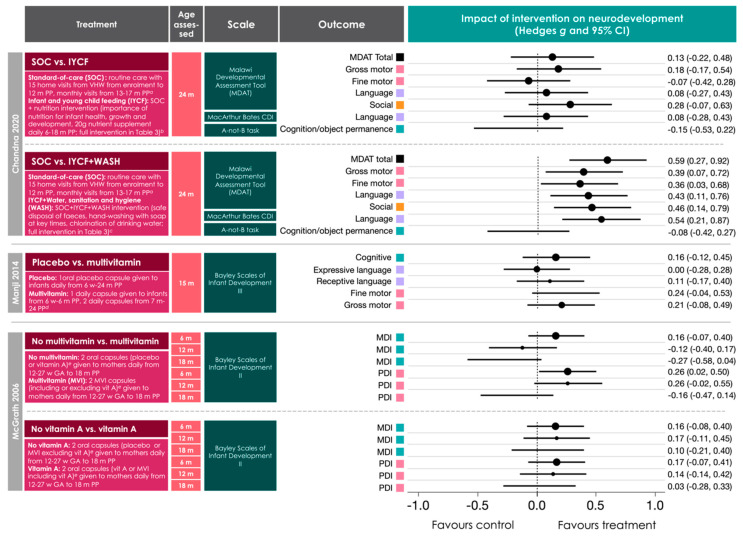
Summary of the results from randomised controls trials investigating the influence of early-life nutrition related interventions on neurodevelopmental outcomes in the first 24 m of life in infants exposed to HIV. Full descriptions of the study treatments or interventions are presented elsewhere (Supplementary Table S3). Chanda et al. (2020) [52] report positive effects of a combined IYCF + WASH intervention on motor, language, and behavioural outcomes at 24 months in a group of 300 infants who are HEU. McGrath (2006) [51] reports positive effects of daily maternal multivitamin supplementation from 12–27 weeks’ gestation to 18 months postpartum on motor developmental outcomes in 327 infants exposed to maternal HIV infant (breakdown of infant HIV status not provided) at six months of age. Methodological quality assessments for these studies are reported in Figure 3. Data are presented in forest plots as Hedge’s *g* (95% CI) in ascending order according to infant age at assessment. SOC = standard-of-care; ICYF = Infant and young child feeding; WASH = water, sanitation, and hygiene; VHW = village health worker; MDAT = Malawi Developmental Assessment Tool; CDI = Communicative Development Inventories; d = days; w = weeks; m = months; PDI = psychomotor development index; MDI = mental development index; CI = confidence interval; HEU = HIV-exposed, uninfected.

Three of the studies included were RCTs that aimed to evaluate the effects of a nutrition-related intervention on neurodevelopmental outcomes in infants exposed to maternal HIV infection and ART [51,52,58]. The interventions are described in full in Supplementary Table S3. Daily maternal multivitamin supplementation (from enrollment at 12–27 weeks’ gestation to 18 months postpartum) associated with higher scores on measures of motor development for infants at 6 months of age, an average increase in motor score of 2.6 points over the 6–18 month period, and reduced risk of motor developmental delay [51]. Daily infant multivitamin supplementation (from 6 weeks to 24 months postpartum) did not associate with any changes in performance on neurodevelopmental assessments performed at 15 months of age [58]. Notably, the mothers of all infants in this cohort also received daily multivitamin supplementation from enrollment to follow up [58]. Infants who received the Infant and Young Child Feeding (IYCF) intervention, which included maternal education on the importance of nutrition for infant health and development and a daily nutrient supplement given to infants from 6 to 18 months postpartum, did not have significantly improved neurodevelopmental outcomes compared to infants who received standard-of-care (SOC; Supplementary Table S3, Figure 5) [52]. However, when the IYCF intervention was given in conjunction with a water, sanitation, and hygiene (WASH) intervention (Supplementary Table S3), infants had higher gross and fine motor, language, and behavioural outcomes at 24 months (Figure 5).

### 3.6. Sex Differences and Neurodevelopmental Outcomes in Infants Perinatally Exposed to Maternal HIV Infection and ART

Few studies ran analyses to examine differences in neurodevelopment for male and female infants. One study reported higher prevalence of “socially withdrawn” classification among female HEU infants (87%) compared to male HEU infants [49]; however, the sample size was small (*n* = 8). Female infants also had higher receptive communication scores than male infants in one population of infants who were HEU [54]. Male infants who were HEU were also more likely to have microcephaly or a diagnosed neurologic condition (not specified) between birth and six months [34]. For female infants who were HEU, the IYCF + WASH intervention associated with higher motor, language, and social scores compared with standard-of-care, while male infants in the IYCF + WASH arm had higher language and social, but not motor scores, compared to SOC. There were no sex-differences for effect of infant multivitamin supplementation on neurodevelopmental outcomes at 15 months [58].

## 4. Discussion

In this formal evidence assessment and meta-analysis, we first aimed to understand how exposure to maternal HIV infection *in utero* and during breastfeeding affects neurodevelopment in infants who are HEI or HEU in the first three years of life. We found that infants who were HEI had poorer neurodevelopmental outcomes in multiple domains in comparison to infants who were HEU or HUU, which is in agreement with previous systematic reviews [12,64]. HEU appears to have persistent, negative effects on neurodevelopment up to age eight [14], albeit to a lesser extent than HEI, and our findings suggest that these negative effects may be detectable within the first three years of life. Our second objective was to investigate how early life nutritional exposures may modify the developmental trajectories of infants exposed to HIV *in utero* or during breastfeeding. We found that the early nutritional environment is a critical contributor to early neurodevelopmental trajectories in infants who have been exposed to HIV *in utero*. Specifically, maternal micronutrient supplementation from 12–27 weeks’ gestation to 18 months postpartum improved motor developmental outcomes at six months in infants exposed to maternal HIV infection, and a comprehensive nutrition intervention, encompassing educational measures and direct nutrient supplementation to infants who were HEU from 6 to 18 months postpartum, improved neurodevelopment across domains when given in conjunction with a WASH intervention [52]. Together, these findings suggest that the perinatal nutritional environment is a modifiable factor that can be optimised to improve child neurodevelopmental outcomes, which has important implications for the rising population of infants who are born HEU [65].

Notably, reports of neurodevelopmental outcomes for infants who were HEU were inconsistent in their findings and are important to discuss within their respective contexts. Five studies that reported little or no differences between HEU and HUU infant groups across multiple domains were all South African cohorts [49,50,53,54,56]. South Africa faces the largest burden of HIV worldwide and has the largest treatment programme globally [66]. In 2018, it was estimated that 87% of women living with HIV who became pregnant had access to ART for PMTCT [67]. The extensive reach of PMTCT efforts in South Africa may contribute to the promising neurodevelopmental outcomes reported for infants who are HEU. How these findings may translate in different contexts, such as Brazil or Botswana, where two studies under review found poorer neurodevelopmental outcomes for infants who are HEU compared to HUU [41,57], remains to be fully understood. Additionally, of the four studies that reported scaled or composite BSID-III scores and were summarised in Figure 4, all but one reporting on cognitive outcomes [41], and all reporting on fine and gross motor, and receptive and expressive language outcomes were South African cohorts [49,50,53]. Notably, there may still be an overall negative effect of HEU on expressive language amongst South African cohorts [49,50], suggesting that HEU may disproportionately affect language development even where PMTCT efforts are extensive, and additional effects to support language developmental trajectories may be necessary.

In studies reporting on sex differences in neurodevelopmental outcomes for infants who are HEU, male infants had poorer performance on measures of receptive communication [54] and were more likely to have microcephaly or a diagnosed neurologic condition between birth and six months [34]. Further, the IYCF + WASH intervention improved motor scores for female, but not male, infants who were HEU [52]. Importantly, few studies overall reported sex-comparisons, and few cohort studies controlled for infant sex in analyses when comparing infant groups. The increased vulnerability of neurodevelopment in male infants when exposed to inflammatory or infectious factors is consistent with previous research [68,69]. As there are notable sex-differences in early brain development [70], and sex-specific differences in breastmilk composition [71,72] and breastfeeding practices [73,74], it is critical that future studies examining relationships between HEU, early life nutrition, and neurodevelopmental outcomes perform sex-based comparisons, present findings stratified by sex, and consider the presence of sex-dependent confounding. Doing so will fill a knowledge gap in whether, and to what extent, the effects of HEU may differentially affect male and female infants.

Notably, two of the studies investigating nutrition-related interventions reported promising effects for infants who are HIV-exposed [51,52]. One of these interventions only improved infant neurodevelopmental outcomes when given in conjunction with a WASH intervention [52]. Addressing WASH may improve infant neurodevelopment by reducing chronic inflammation and improving nutrient absorption in the infant gut [75,76] and should be considered in combination with nutrition-focused efforts to improve development in infants who are HIV-exposed. While infant daily multivitamin supplementation from six weeks to 24 months showed no difference to the placebo group for neurodevelopmental outcomes in one cohort of infants who were HEU [58], all the mothers in this cohort also received daily multivitamin supplementation from enrolment through follow up, which may have improved neurodevelopmental outcomes for this infant group overall. Unexpectedly, only one study reported beneficial associations between breastfeeding and neurodevelopment at 24 months [57], irrespective of infant HIV exposure status, while others reported no associations between breastfeeding practices and neurodevelopment in HEU and HUU infant groups. However, detailed data on length of exclusive breastfeeding [53,56] or mixed feeding [54] were often not reported, limiting our ability to draw conclusions.

The lack of available data on maternal nutrient intakes or levels is a key limitation in the studies reviewed and an opportunity for future research. Maternal nutrient status during pregnancy and the postpartum period is critical to supporting rapid growth and development of the infant [77], and maternal diet in part determines the nutritional composition of breastmilk [78]. Further, how maternal HIV infection and ART may alter breastmilk composition is not well understood. Investigation of breastmilk immune factors among women living with HIV remains limited, and among the few studies that have measured these factors, varied results have been reported. Higher levels of non-specific IgA [79] and IgG [79,80] have been measured in breastmilk from women living with HIV, and higher levels of fucosylated human milk oligosaccharides in breast milk are associated with lower infant mortality among infants who are HEU, but not HEI, during breastfeeding [81]. There is an opportunity to better understand how the benefits of exclusive breastfeeding, including improved maternal health outcomes [82] and constituents in breastmilk [83], could be protective against any adverse effects HEI or HEU on infant neurodevelopment.

Importantly, there are key variables that interact to influence fetal and infant development in pregnancies complicated by maternal HIV infection that were beyond the scope of this review and are important to consider. We did not evaluate relationships between maternal or infant ART and infant neurodevelopment. Previous research has shown that the timing and type of ART initiation for infants who are HIV-exposed may affect neurodevelopmental outcomes [84,85]. Further, as vulnerabilities of the developing fetal brain to the effects of infectious exposures and inflammation vary throughout pregnancy [86,87], the timing of maternal HIV infection, viral suppression, and related antiretroviral treatments is an important consideration, and these data were not frequently available. In 2017, one third of newly transmitted HIV infections in the Middle East and North Africa, eastern Europe, and central Asia occurred in people who injected drugs [65], and neurodevelopmental risks for the fetus and infant related to these comorbid exposures *in utero* are important to understand. Of the studies assessed here, one reported that maternal substance use in pregnancy had a greater (negative) effect on neurodevelopmental trajectories than infant HIV and ART exposure status [47]. Low socioeconomic status is also a known risk factor for poorer cognitive development [88] and is particularly important to consider in the context of HIV infection given the overlaps between HIV/AIDS and economic and food insecurity [9]. Lastly, poor maternal mental health during the pre- and postnatal periods may adversely influence infant neurodevelopmental outcomes [89], and among women living with HIV specifically, depression and mental health vulnerabilities are especially prevalent [90] and have shown to associate with infant health outcomes [91].

Some maternal ART therapies are also associated with increased risk of preterm birth (<37 weeks’ gestation) [92], and preterm birth has independent consequences for infant neurodevelopment [93]. Here, preterm birth was a risk factor for lower cognitive and gross motor scores among infants who were HIV-exposed in one study [55] and among 1400 infants who were HEU in another study [34], those who had diagnosed microcephaly or a neurological condition in the first 6 months of life were more likely to have a lower gestational age at birth. Thus, it is critical to consider how premature birth and *in utero* exposure to maternal HIV/ART may have additive, negative effects for offspring neurodevelopment. Notably, risk of premature delivery among women living with HIV on ART is exacerbated by poor maternal nutritional status [92]. This suggests that interventions to target prenatal maternal nutritional status could improve neurodevelopment in infants who are HIV-exposed through both a reduction in premature birth prevalence, and by the direct effects of improved nutrient resources to support neurodevelopmental processes.

Our ability to draw conclusive statements from this review is limited by the small quantity of published research on this topic. Limiting our inclusion criteria to articles published following the launch of international PMTCT efforts likely contributed to the low number of included articles; however, this was necessary for ensuring comparability and translatability to current day contexts. 

Overall, our review suggests that the early nutritional environment may be leveraged to improve neurodevelopmental trajectories before three years of age for the rising number of infants who are HEU. Further research on how early life nutritional exposures can be enhanced to improve health outcomes for infants who have been exposed to HIV is needed to tailor nutrition-related interventions to match their specific needs. Additionally, our analysis of reported associations between white matter structural abnormalities in brain regions involved in memory, learning, emotional, and cognitive processing [94,95], and performance on neurodevelopmental assessments as early as two to four weeks postpartum, suggests an opportunity for early neurodevelopmental screening to identify infants who may be susceptible to suboptimal developmental outcomes [44]. Early identification of the infants who are at risk of poorer neurodevelopmental trajectories following exposure to HIV, along with an understanding of how the early nutritional environment may be optimised to improve development, are key for determining which infants will benefit from additional support during critical developmental periods in order to mitigate adverse outcome later in life.

## Figures and Tables

**Table 1:** Summary of key findings on neurodevelopmental outcomes from birth to 36 months of age in infants born to mothers living with HIV.

Study	Location	Composition of Cohort by Infant HIV Status	Neurodevelopmental Assessment Tools Used	Age Assessed	Key Findings
Springer 2018 [49]	South Africa	96 (58 HEU, 38 HUU)	- BSID-III - Alarm Distress Baby Scale	12 m	- No differences for average motor, cognitive, language, or behavioural scores between the two infant groups. - More infants who were HEU than HUU had cognitive (five vs. none) or language (28 vs. 18%) developmental delay or decreased vocalisation (25.9 vs. 10.5%). - Seven infants who were HEU (12.1%) vs. one HUU (2.6%) were classified as “socially withdrawn”.
Wu 2018 [42]	China	500 (250 HEU, 250 HUU)	- BSID-III	6–11, 12–17, 18–13, 24–29, or 30–35 m	- HEU associated with lower mean scores and risk of developmental delay in the cognitive and adaptive behaviour domains compared to HUU. - Mean scores in language and motor domains were lower for infants who were HEU compared to HUU, but the difference was not significant. - -Infants who were HEU were more likely to present with below-average language levels than their HUU peers.
da Silva 2017 [41]	Brazil	80 (40 HEU, 40 HUU)	- BSID-III	4, 8, 12, or 18 m	- Cognitive scores (at 8 and 18 m and overall) and motor scores (overall), were lower for infants who were HEU compared to HUU.
Rajan 2017 [40]	India	50 (9 HEI, 41 HEU)	- Developmental Assessment Scale for Indian Infants ^a^	- ^b^	- Across each assessment (1–3 ^b^), average composite scores were lower for infants who were HEI compared to HEU. - All but one (2.4%) infant who was HEU had normal development, while 3/9 (33.3%) infants who were HEI had scores indicating developmental delay in ≥1 assessment(s).
Bass 2016 ^c^ [43]	Uganda	339 (118 HEI, 221 HEU)	- The Mullen Scales of Early Learning - The Color Object Association test	2–5 y	- No differences between the HEI and HEU groups for neurological outcome (included motor and language assessment) or immediate/total recall scores.
Spaulding 2016 [34]	Brazil, Argentina, Mexico, Peru, Bahamas, and Jamaica	1400 HEU	- Head circumference z-score (WHO) ^d^	Birth, 6–12 w, and 6 m	- Microcephaly was observed in 105 infants who were HEU (7.5%), and 134 had at least one neurologic condition (9.6%).
Tran 2016 [44]	South Africa	39 (15 HEU, 24 HUU)	- Diffusion tensor imaging (DTI) - Dubowitz Neurobehavioral Scales	2–4 w	- For whole-brain analysis, there were no significant group differences for diffusion parameters. - Higher fractional anisotropy (FA) was observed in the middle cerebellar peduncle region in infants who were HEU compared to HUU. - Mean diffusivity (MD) and axial diffusivity in the right inferior cerebellar peduncle and left hippocampal cingulum, and MD in the right hippocampal cingulum, were negatively correlated with abnormal neurological signs scores among infants who were HEU. - Abnormal neurological signs scores were positively correlated with FA in the left uncinate fasciculus among infants who were HEU. - HEU associated with higher Dubowitz optimality scores ^e^ compared to HUU.
Hutchings 2014 [45]	Zimbabwe	60 (28 HEI, 32 HEU)	- BSID-III	6 w–12 m	- Infants who were HEI scored lower on measures of cognitive, language, and motor development compared to the HEU group.
Whitehead 2014 [36]	South Africa	56 (27 HEI, 29 HEU; Note c)	- BSID-III	- ^f^	- For language and motor outcomes, infants who were HEI scored lower than HEU at baseline, 3 and 6 m assessments. - Infants who were HEI scores lower than HEU in the cognition domain at the 3 m follow up assessment.
Abubakar 2013 [46]	Kenya	367 (31 HEI, 17 HEU, 319 HUU)	- Kilifi Developmental Inventory - A-not-B task	6–35 m	- Infants who were HEI scored lower on measures of motor, but not cognitive, development than HEU and HUU groups. - Fewer infants in the HEI and HEU groups completed the A-not-B task compared to HUU.
McDonald 2013 ^g^ [37]	Tanzania	311 HE ^h^	- BSID-II	6, 12, and 18 m	- Infant HIV status (HEI) associated with a lower mean PDI and MDI scores compared to HEU.
Kandawasvika 2011 [38]	Zimbabwe	598 (65 HEI, 188 HEU, 287 HUU, 58 HIV-exposed/status unknown)	- Bayley Infant Neurodevelopmental Screener ^i^	3, 6, 9, and 12 m	- Infants who were HEI were twice as likely to exhibit high risk for neurodevelopmental impairment (NDI; 17%) than the HEU (9%) and HUU (9%) groups.
Nso 2009 [39]	Spain	206 HEU	- Not indicated	Birth, 3 and 6 w, 3, 6, 12, and 18 m	- Of the infant sample, 3.64% were classified as having psychomotor developmental delay (compared to a cited estimated population rate of 1.1–2.5%).
Alimenti 2006 [47]	Canada	63 (39 HEU, 24 HUU)	- BSID-II - Vineland Adaptive Behavior Scales	18–36 m	- Infants who were HEU had lower average MDI scores and were more likely to score >1 SD below average in the daily living skills measurements compared to infants who were HUU. - Communication, daily living, socialization, and PDI scores were all lower in the HEU group; however, the differences were not statistically significant.
Tardieu 2005 [48]	France	49 HEU	- Magnetic Resonance Imaging (MRI) - Brunet Lezine scale	26 m (10–44 m)	- Mitochondrial dysfunction was recorded in 22 infants, 16 of whom had abnormal MRI. - The most frequent abnormalities were diffuse hyperintensity in the tegmentum pons (*n* = 9) and the supratentorial white matter (*n* = 9). - Among the 22 infants with mitochondrial dysfunction, 15 had cognitive delay and 6 had motor delay, compared to 5 and 2, respectively, of the 27 infants without mitochondrial dysfunction.

HUU = HIV-unexposed, uninfected; HEU = HIV-exposed, uninfected; HEI = HIV-exposed, infected; m = months; w = weeks; y = years; BSID-III = Bayley Scales of Infant Development 3rd edition [28]; BSID-II = Bayley Scales of Infant Development 2nd edition [59]; PDI = psychomotor development index; MDI = mental development index. ^a^ The Developmental Assessment Scale for Indian Infants assesses motor and cognitive outcomes in one score. ^b^ Development was assessed for all children at enrolment (range 6–18 m) and after 3 months. A third assessment was done for 25 children after 6 months of enrolment. ^c^ Primary outcome-exposure relationships of interest were infant-caregiver interactions and neurodevelopment. Findings from these analyses are not reported here, as they are outside the scope of this review. ^d^ Head circumference was used to ascertain estimates of microcephaly prevalence. ^e^ The Dubowitz optimality score includes assessments for both motor and behavioural function. ^f^ Infants under one year of age were eligible for recruitment. Neurodevelopment was assessed at baseline (prior to initiating Highly Active Antiretroviral Therapy (HAART) for HEI infants), and three and six months after baseline. Breakdown of infant ages at baseline, second, and third assessments was not provided. ^g^ Infant cohort is the same as McGrath et al., 2006. ^h^ A breakdown according to infant HIV status for the 311 infants who had neurodevelopmental assessments not available. All were HIV-exposed. One hundred and thirty-nine HEI and 519 HEU assessments were used in analysis for both cognitive and motor outcomes (repeat measures for infants were included). ^i^ The Bayley Infant Neurodevelopmental Screener includes assessments for both motor and cognitive function in one score.

**Table 2:** Summary of key findings on the influence of nutrition-related factors and interventions on neurodevelopmental outcomes in infants born to mothers living with HIV.

Study	Location	Composition of Cohort Assessed for Neurodevelopment		Neurodevelopmental Assessment Tools Used	Age Assessed	Key Findings
		Infant HIV Status	Treatment Groups ^a^			
Chandna 2020 [52]	Zimbabwe	318 HIV-exposed (6 HEI, 300 HEU, 12 with unknown status)	- SOC: 66 - IYCF: 66 - IYCF + WASH: 103	- Malawi Developmental Assessment tool - MacArthur–Bates Communicative Development Inventory (CDI) - A-not-B task	24 m (range 102–112 w)	- Infants in the IYCF + SOC arm had higher total MDAT, gross motor, language, social, and vocabulary scores compared to SOC infants. - HEU children in the IYCF+SOC arm had higher scores in all MDAT domains and CDI compared to SOC infants.
Springer 2020 [53]	South Africa	59 (27 HUU, 32 HEU)	-	- BSID-III - Strengths and difficulties questionnaire (SDQ)	30–42 m (range)	- No differences for cognitive, language, or motor scores between infants who were HEU vs. HUU, and no child had severe developmental delay in the any of the BSID domains. - Maternal reports of conduct problems were higher for infants who were HUU. - Breastfeeding did not affect neurodevelopmental assessment performance in either group.
Strehlau 2020 [54]	South Africa	49 HEU	-	- BSID-III	12 m	- No infants were classified as having developmental delay, and there were a higher proportion of infants from this cohort whose performance was average, higher average, and superior for cognitive, motor, and language outcomes in comparison to reference norms. - Breastfeeding was initiated at birth for 70% of infants; however, exposure to breastmilk did not associate with performance on neurodevelopmental assessment at 12 m.
Blakstad 2019 ^b^ [55]	Tanzania	453 (206 HEU ^c^, 247 HUU)	-	- BSID-III	15 m (range 14–17 m)	- Daily food expenditure <3000 Tanzanian shillings associated with lower cognitive and fine motor outcomes in HIV-exposed infants ^d^, and with lower fine motor ^e^ and expressive language outcomes in HUU infants ^f^. - Maternal reports of meat consumption >4 times/w associated with higher cognitive scores in HEU infants, and consumption 0–3 or >4 times/w in HUU infants associated with higher cognitive scores. - Stunting ^g^ was associated with low language scores (HEU) and low fine motor scores (HUU) ^h^.
Wedderburn 2019 [50]	South Africa	6 m: 260 (61 HEU, 199 HUU)24 m: 732 (168 HEU, 564 HUU)	-	- BSID-III	6 and 24 m	- No differences between groups for cognitive, language, or motor scale scores at 6 m. - Infants who were HEU had lower mean scores on the cognition scale at 24 m compared to HUU, although this difference lessened in adjusted analyses. - HEU associated with lower scores and higher odds of delayed development in expressive and receptive language domains at 24 m compared to HUU in both unadjusted and adjusted analyses. - <20% of mothers exclusively breastfed for 6 m, and exclusive breastfeeding duration was shorter for infants who were HEU. - Exposure–outcome relationships did not change after adjusting for length of exclusive breastfeeding, or exclusive breastfeeding to 6 months vs. not.
Le Roux 2018 [56]	South Africa	521 (306 HUU, 215 HEU)	-	- BSID-III	13 m (range 12–14)	- Average scores did not differ between HEU and HUU groups for cognitive, motor, or language domains. - Infants who were HEU were twice as likely to have cognitive developmental delay compared to HUU in both crude and adjusted models. - Associations between HEU and increased risk of motor delay (compared to HUU) were identified in crude models but attenuated in adjusted models. - Duration of any breastfeeding was shorter for infants who were HEU compared to HUU (6 vs. 10 m) and did not associate with risk of developmental delay for HEU vs. HUU.
Chaudhury 2017 [57]	Botswana	724 (387 HUU, 337 HEU)	-	- BSID-III - Developmental Milestones Checklist	24 m	- Infants who were HEU had higher scores in personal–social and cognitive domains, compared to HUU after adjusting for key covariates. - In crude analyses, HEU associated with lower expressive language scores, but the differences were attenuated in adjusted analysis. - Breastfeeding was more common among infants who were HUU compared to HEU (99.5 vs. 9%), while food insecurity was more common among HEU (mild, moderate, or severe: 71.8 vs. 59.4%). - Infants who were ever breastfed had higher cognitive and expressive language scores at 24 m compared to infants who were never breastfed, and food insecurity associated with lower gross motor scores, irrespective of HIV status.
Manji 2014 [58]	Tanzania	192 HEU ^b^	- Placebo: 99 - MVI: 93	- BSID-III	15 m (range 14–17 m)	- Infant multivitamin supplementation did not lead to improved neurodevelopmental outcomes cognitive, motor, or language domains.
McGrath 2006 [51]	Tanzania	327 HE ^i^	- No MVI: 158 - MVI: 169 - No Vitamin A: 147 - Vitamin A: 180	- BSID-II (Mental Development Index (MDI); Psychomotor Development Index (PDI))	6, 12 and 18 m	- Maternal multivitamin supplementation associated with improved motor scores at 6 m, an average increase in PDI score of 2.6 points over the 6 to 18 m period, and reduced risk of motor delay. - Maternal multivitamin supplementation did not affect MDI scores, and maternal vitamin A supplementation did not affect MDI or PDI scores.

HUU = HIV-unexposed, uninfected; HEU = HIV-exposed, uninfected; HEI = HIV-exposed, infected; m = months; w = weeks; BSID-III = Bayley Scales of Infant Development 3rd edition [28], BSID-II = Bayley Scales of Infant Development 2nd edition [59]. ^a^ Detailed descriptions of the nutrition-related interventions are provided in Supplementary Table S3. ^b^ Reports on two infant cohorts who were enrolled in two separate micronutrient trials in Tanzania, including the same cohort as Manji et al., 2014. ^c^ Manji et al. (2014) [58] report neurodevelopmental findings for 192 infants who were HEU (of 206 HIV-exposed infants total, 14 who tested positive for HIV at 6 weeks of age and were excluded from analyses). Blakstad et al. (2019) [55] report that in this cohort, all 206 HIV-exposed infants were HEU at 15 months of age. ^d^ After adjusting for child sex, child age, and BSID-III examiner. ^e^ After adjusting for marital status, maternal height, maternal education, household size, food expenditure, child sex, child age, and BSID-III examiner. ^f^ After adjusting for prior pregnancies, maternal height, maternal body mass index, food expenditure, meat consumption, child sex, child age, and BSID-III examiner. ^g^ Stunting is defined as length-for-age z-score < −2 SD below the median and is a marker of chronic undernutrition (WHO Multicentre Growth Reference Study Group, 2006). ^h^ After adjusting for maternal education, daily expenditure on food, child sex, age, and BSID-III examiner. ^i^ Authors did not report cohort breakdown according to infant HIV status but did report that infant HIV status did not modify relationships between multivitamin supplementation and neurodevelopmental outcomes.

## Supplementary Materials

The following are available online at https://www.mdpi.com/2072-6643/12/11/3375/s1, Table S1: PRISMA Checklist, Table S2: Methodological quality assessment criteria set a priori, Table S3: Descriptions of trial interventions for randomized control trials on early life nutrition-related factors and neurodevelopment in infants exposed to HIV, Figure S1: Evidence-based review keyword search terms, Figure S2: PRISMA flow diagram for article selection, Figure S3: Locations of studies considered in review, Figure S4: Funnel plot with effect estimates and standard error for meta-analysis of cognitive sub-scale composite and scaled scores (Bayley Scales of Infant Development, 3rd ed.) from four studies (7 cohorts).
