# Supplementary material for: In Utero HIV Exposure and the Early Nutritional Environment Influence Infant Neurodevelopment: Findings from an Evidenced Review and Meta-Analysis
Source: Nutrients. 2020 Nov 2;12(11):3375. doi: 10.3390/nu12113375 (PMC7692402; doi:10.3390/nu12113375)
Supplement: Supplementary file 1 [file nutrients-12-03375-s001.zip › HIV neuro nutrition review Nutrients Supplementary files final PDF/supplmentary figures HIV neuro nutrition review ms v4 Nutrients.pdf]

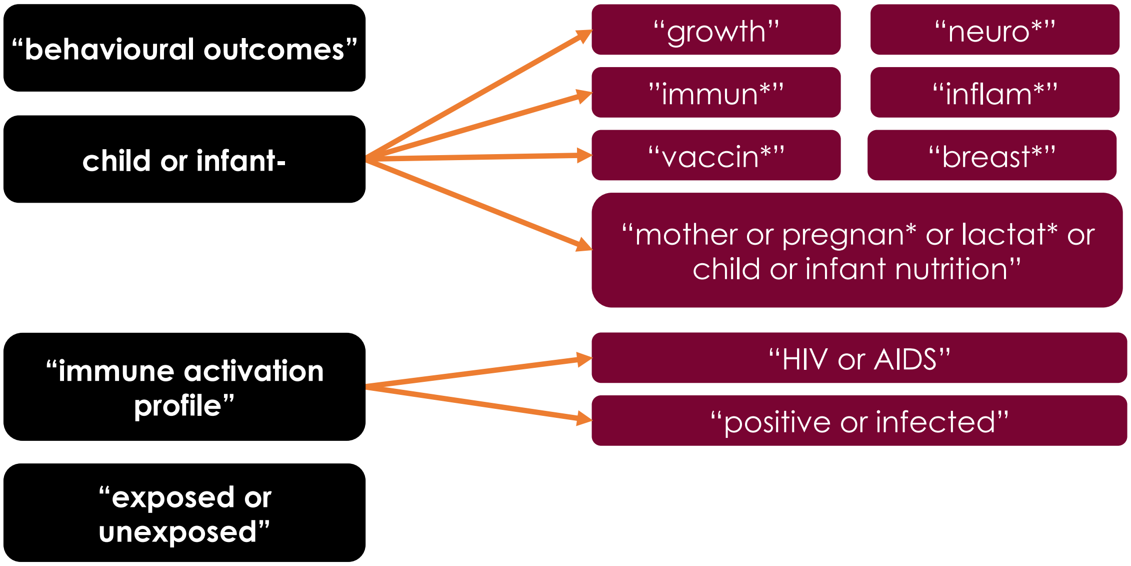

Supplementary Figure 1. Evidence-based review keyword search terms.

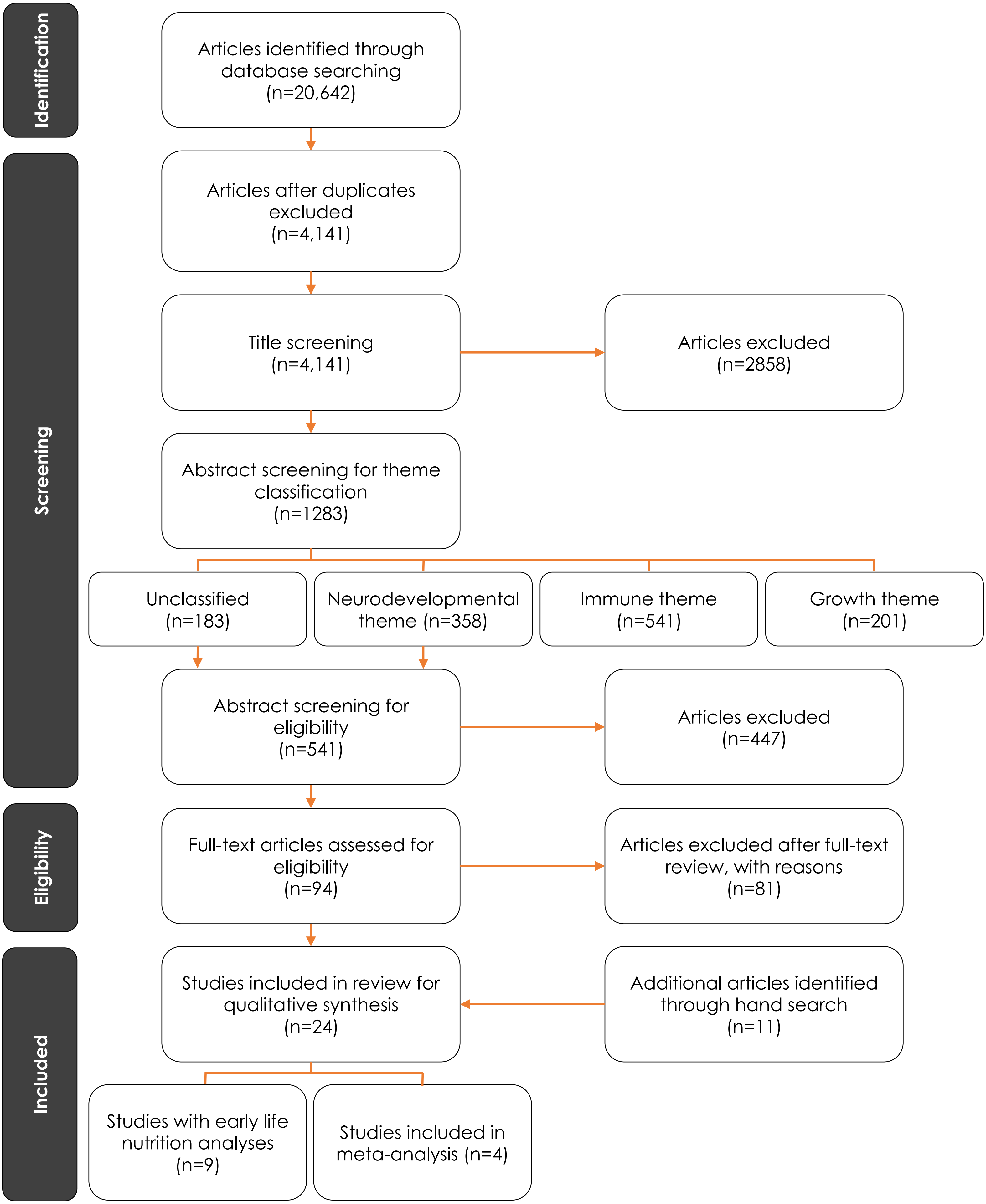

Supplementary Figure 2. PRISMA flow diagram for article selection. Additional search by hand was performed on March 25, 2020 using the same set of pre-determined key words in PubMed, CINAHL, ProQuest, and Web of Science.

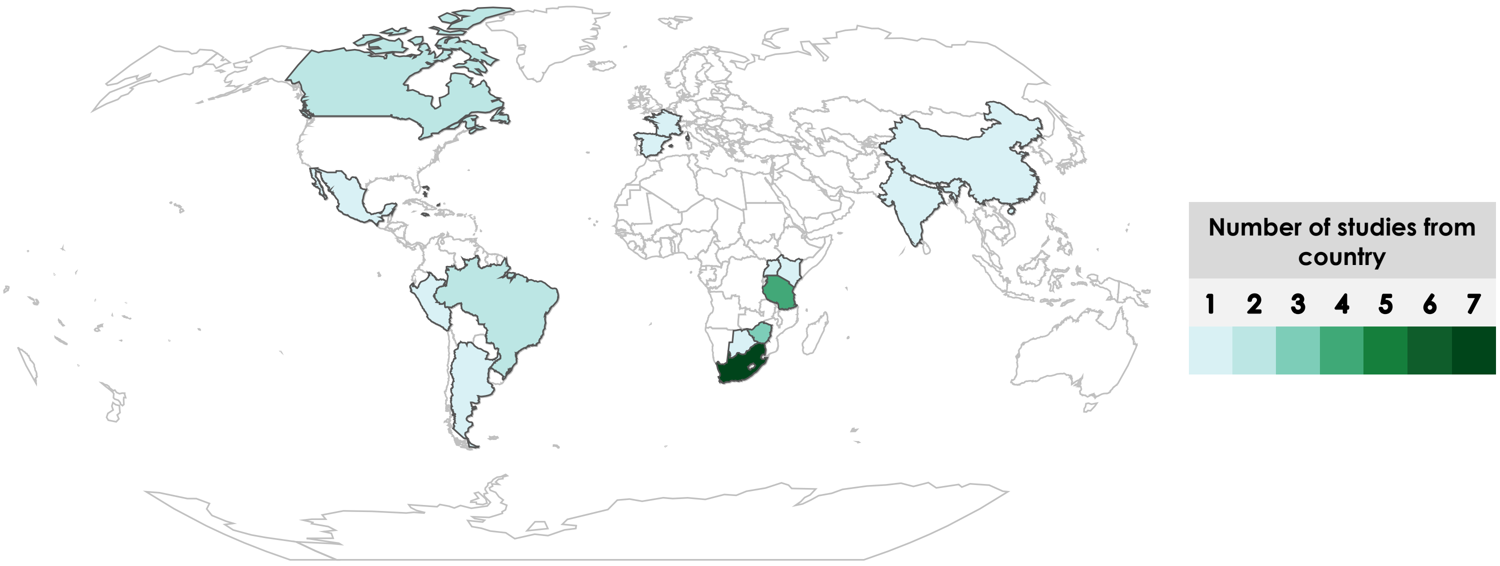

Supplementary Figure 3. Locations of studies considered in review. South Africa (n=7) had the highest representation in studies under review. 57% (n=17) of the cohorts included in studies under review were from Africa, followed by 17% (n=5) from North America, 13% (n=4) from South America, and 7% (n=2) from both Asia and Europe. One study report on data from cohorts in Brazil, Argentina, Peru, Mexico, Bahamas, and Jamaica (Spaulding et al., 2016). For the purpose of this figure, each of these cohorts was counted once.

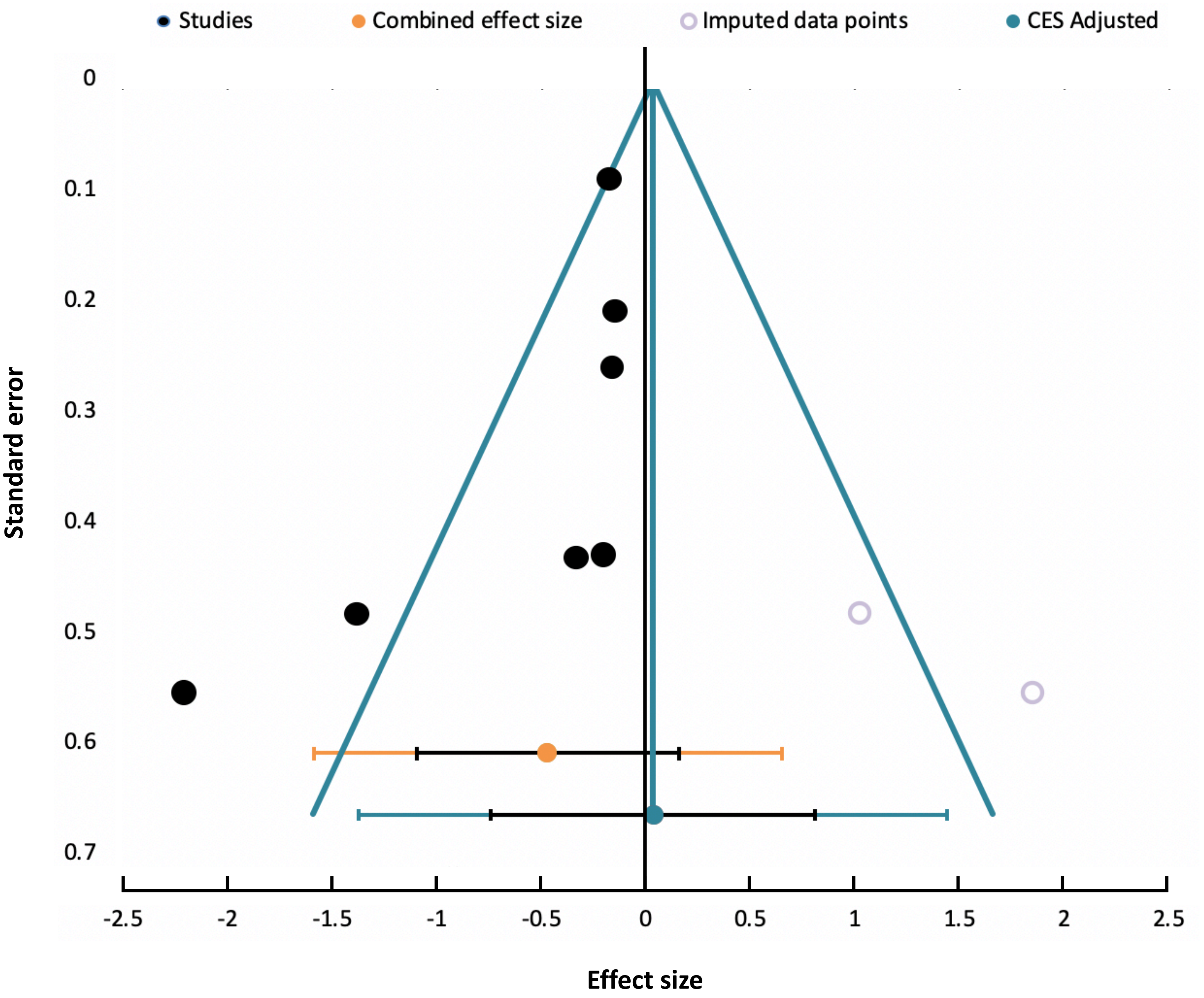

Supplementary Figure 4. Funnel plot with effect estimates and standard error for meta-analysis of cognitive sub-scale composite and scaled scores (Bayley Scales of Infant Development, 3rd ed.) from four studies (7 cohorts). CES = Combined effect size.
