# Supplementary material for: In Utero HIV Exposure and the Early Nutritional Environment Influence Infant Neurodevelopment: Findings from an Evidenced Review and Meta-Analysis
Source: Nutrients. 2020 Nov 2;12(11):3375. doi: 10.3390/nu12113375 (PMC7692402; doi:10.3390/nu12113375)
Supplement: Supplementary file 1 [file nutrients-12-03375-s001.zip › HIV neuro nutrition review Nutrients Supplementary files final PDF/supplementary Tables HIV neuro nutrition review ms v6 Nutrients.pdf]

## Supplementary Table 1. PRISMA Checklist [19].

| Section/topic | # | Checklist item | Reported on page # |
|---|---|---|---|
| **Title** | | | |
| Title | 1 | Identify the report as a systematic review, meta-analysis, or both. | 1 |
| **Abstract** | | | |
| Structured summary | 2 | Provide a structured summary including, as applicable: background; objectives; data sources; study eligibility criteria, participants, and interventions; study appraisal and synthesis methods; results; limitations; conclusions and implications of key findings; systematic review registration number. | 1 |
| **Introduction** | | | |
| Rationale | 3 | Describe the rationale for the review in the context of what is already known. | 1-3 |
| Objectives | 4 | Provide an explicit statement of questions being addressed with reference to participants, interventions, comparisons, outcomes, and study design (PICOS). | 3-4 |
| **Methods** | | | |
| Protocol and registration | 5 | Indicate if a review protocol exists, if and where it can be accessed (e.g., Web address), and, if available, provide registration information including registration number. | N/A |
| Eligibility criteria | 6 | Specify study characteristics (e.g., PICOS, length of follow-up) and report characteristics (e.g., years considered, language, publication status) used as criteria for eligibility, giving rationale. | 3-5 |
| Information sources | 7 | Describe all information sources (e.g., databases with dates of coverage, contact with study authors to identify additional studies) in the search and date last searched. | 3-5 |
| Search | 8 | Present full electronic search strategy for at least one database, including any limits used, such that it could be repeated. | Supplemental figure 1 |
| Study selection | 9 | State the process for selecting studies (i.e., screening, eligibility, included in systematic review, and, if applicable, included in the meta-analysis). | 3-5 |
| Data collection process | 10 | Describe method of data extraction from reports (e.g., piloted forms, independently, in duplicate) and any processes for obtaining and confirming data from investigators. | 3-5 |
| Data items | 11 | List and define all variables for which data were sought (e.g., PICOS, funding sources) and any assumptions and simplifications made. | 3-5 |
| Risk of bias in individual studies | 12 | Describe methods used for assessing risk of bias of individual studies (including specification of whether this was done at the study or outcome level), and how this information is to be used in any data synthesis. | 4-5 |
| Summary measures | 13 | State the principal summary measures (e.g., risk ratio, difference in means). | 7-10 |
| Synthesis of results | 14 | Describe the methods of handling data and combining results of studies, if done, including measures of consistency (e.g., $I^2$) for each meta-analysis. | 5 |

**Supplementary Table 2.** Methodological quality assessment criteria set a priori.

| Checklist item requiring specification | Criterion |
|---|---|
| **Newcastle-Ottawa Quality Assessment scale (cohort studies) [24]** | |
| Comparability | 1. Determined based on whether the authors controlled for infant sex and age at assessment in analyses |
| Was follow-up long enough for outcome to occur? | 1. As neurodevelopment within the first three years of life was the outcome of interest, demonstration that outcome of interest was not present at the start of the study was not required and all articles received a ‘yes’ assessment for this criterion. |
| Adequacy of follow up cohorts | 1. Where cohorts had cross-sectional neurodevelopmental data, adequacy of follow up cohorts was not assessed;<br>2. Where neurodevelopmental data were longitudinal, adequacy of follow up cohorts was considered where subjects lost to follow up were minimal (<20%) or analyses were run to establish similarity between infants retained at follow up vs. not. |
| **Quality Appraisal Tool for Case Series (18-item checklist) [25]** | |
| Are characteristics of the participants included in the study described? | 1. Characteristics of the cohort that were important to include were pre-defined as: number of participants (infants), age range of infants with neurodevelopmental assessment and infant sex. |
| Did participants enter the study at a similar point in the disease? | 1. Based on our outcomes of interest, this was redefined as “Did participants enter the study at a similar point in development (i.e., age)”. |
| Was the intervention clearly described in the study? | 1. “Intervention” was modified to be “exposure” of interest, and defined as: Maternal HIV infection and information on ART;<br>2. A point was given if authors reported details on whether or not the mothers were on ART and what ART treatments mothers were on. |
| Were additional (co-interventions) clearly described in the study? | 1. “Co-intervention” was modified to be “co-exposure” of interest, and defined as: infant HIV exposure status and information on ART;<br>2. A point was given if authors reported details on whether or not an infant contracted HIV infection prior to or during the study, or received ART intervention |
| Are adverse events reported? | 1. Adverse events were not considered as no intervention was being employed. |
| **Cochrane Collaboration’s Tool for Assessing Risk of Bias [26]** | |
| Other bias | 1. Defined as assessment of compliance to intervention |

**Supplementary Table 3.** Descriptions of trial interventions for randomized control trials on early life nutrition-related factors and neurodevelopment in infants exposed to HIV.

| Study | Intervention level | Description of intervention |
|---|---|---|
| Chandna 2020 | Standard-of-care (SOC) | - "Promotion of exclusive breast feeding to 6 months, uptake of antenatal and neonatal care, PMTCT, immunisations, family planning."(Chanda et al., 2020, pg. 2) |
| | Infant and young child feeding (IYCF) | - "All SOC messages plus (1) importance of nutrition for infant health, growth and development; (2) feeding nutrient-dense food and 20g small-quantity lipid-based nutrient supplement (SQ-LNS; Nutriset, Malaunay, France) daily from 6 to 18 months; (3) processing locally available foods to facilitate mastication and swallowing; (4) feeding during illness; (5) dietary diversity. Monthly delivery of SQ-LNS from 6 to 18 months." (Chanda et al., 2020, pg. 2) |
| | IYCF + Water, sanitation and hygiene (WASH) | - All SOC and IYCF interventions "plus (1) safe disposal of faeces; (2) hand-washing with soap at key times; (3) protection of infants from geophagia and animal faeces ingestion; (4) chlorination of drinking water and (5) hygienic preparation of complementary food. Ventilated improved pit latrine constructed within 6 weeks of enrolment; two hand-washing stations; plastic mat and play yard; monthly delivery of soap and chlorine (WaterGuard, Nelspot, Zimbabwe)." (Chanda et al., 2020, pg. 2) |
| Manji 2014 | Infant placebo supplementation | - One placebo capsule daily from 6 weeks to 24 months. Identical in taste and appearance to the active capsule. |
| | Infant multivitamin supplementation | - One capsule daily from age 6 weeks to 6 months of: 60 mg of vitamin C, 8 mg of vitamin E, 0.5 mg of thiamine, 0.6 mg of riboflavin, 4 mg of niacin, 0.6 mg of vitamin B6, 130 ug of folate and 1 mg of vitamin B12. From 7 months to the end of follow up, 2 capsules given daily. All mothers were provided with oral multivitamins from enrollment to the end of follow up. |
| McGrath 2006 | No maternal multivitamin supplementation | - 2 daily tablets of vitamin A (30 mg of β-carotene plus 5000 IU preformed vitamin A) from 12-27 weeks' gestation to 18 months postpartum or;<br>- 2 daily tablets of placebo from 12-27 weeks' gestation to 18 months |
| | Maternal multivitamin supplementation | - 2 daily tablets of multivitamins excluding vitamin A (20 mg of $B_1$, 20 mg of $B_2$, 25 mg of $B_6$, 100 mg of niacin, 50 μg of $B_{12}$, 500 mg of C, 30 mg of E, and 0.8 mg of folic acid) from 12-27 weeks' gestation to 18 months or;<br>- 2 daily tablets of multivitamins including vitamin A from 12-27 weeks' gestation to 18 months |
| | No maternal vitamin A supplementation | - 2 daily tablets of multivitamins excluding vitamin A from 12-27 weeks' gestation to 18 months or;<br>- 2 daily tablets of placebo from 12-27 weeks' gestation to 18 months |
| | Maternal vitamin A supplementation | - 2 daily tablets of vitamin A from 12-27 weeks' gestation to 18 months or;<br>- 2 daily tablets of multivitamins including vitamin A from 12-27 weeks' gestation to 18 months |
